# *‘We’re More Prepared than Before*: Understanding the Strategies Used by a Non-governmental Organization During the COVID-19 Pandemic in Sub-Saharan Africa

**DOI:** 10.1177/2752535X251317651

**Published:** 2025-01-31

**Authors:** Satveer Dhillon, Isaac Luginaah, Susan J. Elliott, Justine Nagawa, Ronah Agaba Niwagaba

**Affiliations:** 1 Department of Geography and Environment, 6221 Western University, London, ON, Canada; 2 Department of Geography and Environmental Management, 8430 University of Waterloo, Waterloo, ON, Canada; 3 Reach One Touch One Ministries, Mukono, Uganda

**Keywords:** COVID-19 pandemic, public health crises, social resilience, Sub-Saharan Africa, NGOs

## Abstract

**Introduction:**

The COVID-19 pandemic had a negative impact on populations worldwide, particularly on older adults residing in low - and middle-income countries. Due to these negative impacts, non-governmental organizations (NGOs) provided extensive support, which affected their operations.

**Methods:**

Using the social resilience framework, the purpose of this study was to better understand what strategies NGOs used to support vulnerable populations and how they are building back stronger from the COVID-19 pandemic. In the fall of 2022, 26 (virtual) in-depth interviews were conducted with staff and volunteers from an NGO supporting older adults in Uganda.

**Results:**

Several key themes emerged including using existing resources to better support older adults and staff and the importance of having multiple sources of revenue to support organizational operations.

**Discussion:**

The key lessons learned by NGO staff and volunteers can be utilized to enact policy and practice change to help strengthen NGOs’ social resilience. This would allow them to continue implementing innovative strategies to support vulnerable populations during times of crisis.

## Introduction

The COVID-19 pandemic was an unprecedented public health crisis that impacted countries and communities worldwide.^1,2^ One group of individuals that were significantly impacted was older adults (aged 60 years or above).^3^ Indeed, studies show that more than 80% of COVID-19 deaths occurred among older adults.^4^ Further, not only were they more susceptible to the virus, but public health restrictions also had consequences for older adults, especially for those residing in low- and middle-income countries (LMICs). For example, within Sub-Saharan Africa (SSA), lockdowns negatively impacted older adults’ livelihoods, further exacerbating food insecurity.^5–7^

As a result of the far-reaching impacts of the COVID-19 pandemic, non-governmental organizations (NGOs) stepped up their activities to provide support alongside the government. An NGO is an organization independent of the government that conducts activities to provide essential services and undertakes community development.^8,9^ While the academic discourse has criticisms of NGOs, including issues surrounding accountability, erosion of government obligations, issues of autonomy and their actual effectiveness,^10,11^ it cannot be denied they are utilized extensively during public health emergencies. For example, in Iran, several NGOs mobilized resources and provided mental health services to better support the community during the COVID-19 pandemic.^12^ Also, in some regions worldwide, individuals may have decreased trust in the government, further increasing their dependence and reliance on NGOs.^13^ Historically, NGOs have continuously provided care during times of crisis in SSA. For example, during Ebola, more prominent international NGOs, such as Doctors Without Borders, helped strengthen health care infrastructure and provide medical supplies to patients.^14^ NGOs also implemented several programs during the HIV/AIDs epidemic.^15^

However, the extensive dependence on NGOs during the pandemic has impacted their operations. For example, NGOs in SSA experienced increased demand and a reduction in funding, impacting capacity and service delivery.^16^ With the potential threat of another public health crisis, it is imperative to learn what strategies NGOs used during the COVID-19 pandemic to support particularly vulnerable populations and how they are recovering from the negative impacts of the pandemic. These insights can be used by other NGOs both inside and outside SSA to help develop strategies to provide improved support to vulnerable communities and build back stronger after public health crises.

This paper presents the results of a qualitative investigation of the strategies used by an NGO in Uganda during the COVID-19 pandemic to support older adults and how they are building back stronger.

This work is informed by the social resilience framework. Social resilience is the ability of groups and institutions to maintain system function when responding to significant disturbances such as the COVID-19 pandemic or other disasters.^17,18^ Furthermore, the concept of social resilience also allows for identifying opportunities for innovation and development during times of crisis.^19^ Scholars have suggested that there are three different types of capacities within this framework: coping capacity, measured by how people overcome immediate threats by using directly available resources and skills^18,20^; adaptive capacity, which is the ability to use preventive or mitigation strategies based on past learnings^21,18^; and transformative capacity, defined as the ability to access assistance from the wider social-political environment.^18,22^ In this sense, our research will utilize the social resilience framework to better understand the strategies employed by an NGO during the COVID-19 pandemic.

The paper is organized into five sections. The first section is the introduction, followed by the research design and methods section, which further explains the methods used to address the research objectives. The following section, results, highlights the results as they align with our research objectives and the social resilience framework. The discussion section places the findings of the research within the current literature while explaining the contributions of the research. The final section presents the conclusions, potential limitations of the research and areas for future research.

### Study Context

Uganda is a country within SSA, located in east-central Africa, with a population of 48.6 million.^23,24^ The population of older persons is expected to increase significantly in Uganda. Indeed, in 2020, the number of older persons was 1.5 million, and by 2050, that number is projected to increase to 6 million.^25^ Exacerbated by the lack of pensions and access to education and healthcare, older adults often have to rely on NGOs to help meet their daily needs.^26–29^ The dependence on NGOs is further highlighted by the increase in NGOs from 160 to 14,000 in Uganda.^30^

Reach One Touch One Ministries (ROTOM) is an example of an NGO that provided extensive support during the COVID-19 pandemic. ROTOM was founded in 2004 in Uganda to help support the older adult population. At this time, ROTOM has four international partners (Canada, USA, Germany, UK) that are responsible for providing support to both ROTOM Uganda and ROTOM Ethiopia. ROTOM provides support to older adults and their dependents by providing them with safe water and sanitation, health care, housing, food supplies and educational access for their grandchildren.^31^

## Research Design and Methods

Interviews were conducted with NGO staff and volunteers (*n* = 28) virtually using Zoom, employing a qualitative descriptive design to explore the strategies used by an NGO in SSA to support older adults during the COVID-19 pandemic and the strategies implemented to build back stronger from the pandemic. Participants must have been eighteen years or older and have been working or volunteering with the NGO during the COVID-19 pandemic. Using theoretical sampling, participants were recruited by sharing information about the research through a letter of information and a recruitment poster circulated within the NGO.

All the participants interviewed held various roles, such as being a village volunteer or assisting with special projects (Table 1). The role of the village volunteers was to provide on-the-ground support for ROTOM-related activities. Field officers were ROTOM staff members who were responsible for providing updates to the ROTOM leadership team and for coordinating ROTOM-related activities.

**Table 1.** Roles of Interviewees in ROTOM.

Role	Number (%)
Leadership (Board Members, Senior Staff, Executive Directors)	7 (27%)
Village Volunteers	6 (23%)
Healthcare (Doctors, Nurses)	4 (15%)
Special Projects (Agriculture Initiatives, ROTOM School)	4 (15%)
Field Officer	3 (11%)
Evangelist	2 (8%)
TOTAL	26 (100%)

During the recruitment phase, potential participants were provided a letter of information that explained the research objectives and potential risks and benefits. From September 2022 to December 2022, all interviews were conducted in English through Zoom. The interviews began with an overview of the letter of information and consent process and all participants were provided an opportunity to ask questions before the recording began. An interview guide was used to understand the strategies implemented during the COVID-19 pandemic to support older adults in SSA and how the NGO is recovering from the impacts of the pandemic. Topics included the key lessons learned from the COVID-19 pandemic, the support received from other entities and the programs that were implemented to support the older adult population. Interviews ranged from 19 minutes to 1 hour and 5 minutes, with the average being 36 minutes. Interviews were conducted until data saturation, which is when collecting additional data does not provide any new insights.^32,33^ During the interviews, common themes began to emerge from the participants including the importance of being prepared for future crises and how essential it is to follow public health measures. Once these themes consistently began to appear, we knew data saturation occurred.

All interviews were transcribed verbatim and anonymized in Microsoft Word and then uploaded into NVivo for thematic analysis. For thematic analysis, a codebook was developed using deductive and inductive coding to assist with line-by-line coding of the transcripts. The initial codebook was developed based on examples from previous interview guides used in similar research projects, academic literature and the interview questions. To ensure consistency, the transcripts and themes were reviewed several times. In order to further validate the data, the results were shared with all of the participants to provide them with the opportunity to review the results and add additional information.^34^ Of 26 individuals, six individuals provided comprehensive feedback. Results are presented in the next section, organized around three central themes.

This study was conducted following ethical practices. Verbal informed consent was obtained from each participant before the interviews began, and all interviews were audio recorded with the participants’ permission. Participants were informed of confidentiality practices, such as the omission of their names in the transcription process. Also, participants were reminded that they had the right to refuse to answer questions and could stop the interview at any time. Ethical clearance was provided by Western University’s Non-Medical Research Ethics Board (Project ID: 121196). Informed verbal consent was provided by all participants.

As researchers, we recognize that our backgrounds and prior experiences may have influenced various parts of the research process. To mitigate the potential impact of these influences, we actively collaborated with the NGO throughout the research process. This included employing the method of member checking to ensure the results were rooted in the participants’ experiences.

## Results

These results are organized around three emerging themes, informed by the core components of social resilience (coping, adaptive capacity and transformative capacity): (1) the NGO’s coping capacity to support older adults as well as the staff and volunteers themselves; (2) utilizing adaptive capacity to build back stronger and (3) using transformative capacity from other entities. Table 2 provides an overview of the results.

**Table 2.** Summary of the Results Using the Social Resilience Framework.

Components of social resilience	Examples
Coping	• Providing COVID-19 vaccines and personal protective equipment
	• Following public health measures
	• Mobilizing volunteers
	• Allowing staff and volunteers to work from home when possible
Adaptive	• Improving sustainability through implementing a reserve fund and having different sources of funding
	• Strengthening health facilities
Transformative	• Further support from government required
	• Support from international donors

### Coping Capacity

Several strategies were implemented to support the older adult population to help manage the immediate impacts of the COVID-19 pandemic. One of the most important initiatives to reduce the severe consequences of the virus was making COVID-19 vaccinations available. Participants stated that there was high vaccine uptake amongst the older adults supported by ROTOM, as over 90% of them received vaccinations. There were several reasons for this high vaccine uptake, including the trust older adults had in the NGO as highlighted by a field officer:

*So it was not very easy to convince people to come for the vaccine. However, the relationship we had with all the people for a long time. ROTOM has been serving these people for over 19 years. They had trust in us. Most of them say that if it was not ROTOM, I wouldn't have gotten vaccinated *(P1 NGO Uganda Field Officer).

The NGO staff and volunteers illuminated the importance of following public health measures and providing personal protective equipment, especially to vulnerable populations. Older adults were taught how to wash their hands and the importance of social distancing. In terms of hand washing, older adults could access safe water through water tanks and containers. They were also provided with essential items such as soap and face masks:

*So, we started giving out soap to each senior every month. It was really helpful during that time. We also ordered face masks. At least everyone had a pair or two or three masks. They had washable masks that we ordered for them, and they would wash one and put on the other *(P2 NGO Uganda Field Officer).

The NGO also implemented Village Health Teams, comprised of volunteers living in the villages, to support older adults. These teams would be responsible for providing COVID-19-related information to older adults and ‘*taking the services closer to them*.*’* Through this team, nurses and volunteers delivered medications and healthcare to the older adults’ homes, which is essential as the pandemic ‘*created a gap between the healthcare providers and the seniors*.*’* Volunteers on the Village Health Teams were also taught how to conduct simple health tests when they went to visit the older adults:

*We trained volunteers who acted as Village Health team volunteers, who later on helped us when visiting seniors in their homes. We told them how to take blood pressure measurements and how to record them […] And so we conducted the teachings, and also we had to always follow up with them on a daily basis to check on how the seniors were doing […]. When we had a total lockdown, and there was no movement at all, we had to visit the seniors, which was authorized by the government, and pick up a senior in case they were sick. The other thing we did was carry out monthly refills of medicines for those seniors with chronic illnesses like hypertension, diabetes, and COPD* [chronic obstructive pulmonary disorder – a common respiratory illness particularly among older women due to the conditions related to cooking]*, among others. So we could ensure that they get their medicines delivered to them in their homes so that they still continue to take their medicines* (P_12_ NGO Uganda Healthcare).

The new approach of Village Health Teams has also been described as *‘a positive*, *it’s a blessing these days’* by the NGOs’ healthcare team. The Village Health Team also provided social support and motivation to older adults. Older adults were also taught how to wear masks and wash their hands.

Another interesting innovation was encouraging older adults to use radios, that were provided to them a few years ago by the NGO, to hear updated information on the COVID-19 pandemic:

*Everyone has a radio. They might not afford a TV, but every senior has a radio. And the radio would always talk about the pandemic. If you didn’t get a chance to learn from community volunteers, you can learn from the radio *(P10 NGO Uganda Healthcare).

The NGO also implemented several strategies to support staff and volunteers, beyond the provision of masks and sanitizers for protection. The staff and volunteers worked from home during this time and due to technology, they could complete various tasks as described by a staff member:

*We could send emails, we could make calls, we could Zoom, we could transfer money using from one phone to another. […] So basically, what we learned most during the COVID-19 pandemic is how to use technology to perform our duties* (P_1_ NGO Uganda Field Officer).

### Adaptive Capacity

While coping strategies helped the NGO to address immediate challenges during the COVID-19 pandemic, the NGO staff and volunteers highlighted many strategies they felt would be useful to build resilience and recover from the pandemic, which is the focus of adaptive capacity. Firstly, it is imperative to ensure that the organization is sustainable, and one way to do so is to build a reserve fund, as stated by several participants:

*So that means any time, anything can happen and that was an eye-opener to us […]. There's a need for an emergency fund so that in case of such emergencies, at least we are able to move on. Of course, we moved on, but going forward, we need, I think, a more sustainable strategy so that in case of other conditions in future, we are able to move on* (P_13_ NGO Uganda Special Projects).

On a smaller scale, several participants stated the importance of individuals saving money and being prepared for future crises. The NGO staff and volunteers have also increased their support for food security by encouraging older adults to build gardens at their homes.

Another adaptive capacity recommendation is the need to strengthen health facilities, as reported by several NGO staff and volunteers. The NGO, in response to the COVID-19 pandemic, has built a new isolation room, increasing its medical capacity while also purchasing more equipment, including oxygen tasks. As a result, a healthcare team member stated, ‘*we’re more prepared than before when we were hit*.*’*

Finally, the NGO staff and volunteers echoed the importance of remaining vigilant in case another epidemic or pandemic occurs:

*Then another thing that ROTOM is doing differently, of course, is that we are continuing to train our grandmothers and seniors to take care of their health. To make sure that they should not stop washing hands and keeping clean to take care of their entire health because, when one’s health is taken care of, the risks of diseases are much more reduced. And again, as ROTOM, we continue to see a need of continuing to support our beneficiaries with soap and even clean water […]. Many seniors have been supported with water tanks to be able to get clean and safe water, and every month, they receive soap to help with proper hygiene* (P_13_ NGO Uganda Special Projects).

At the time of the interviews, Ebola was a concern, and the COVID-19 pandemic helped them prevent the spread of Ebola due to the preventative actions taken.

Further, it was recommended that the government should connect with local organizations ‘*because the government alone cannot work on such issues.*’

### Transformative Capacity

The adaptive strategies highlighted by the NGO staff and volunteers laid the groundwork for change, however by focusing on transformative capacities, the NGO staff and volunteers underscored several ways to create long-lasting shifts. For example, the NGO typically receives two types of financial support: from the government and international donors. There was also an underlying theme emphasizing the need for fundamental, transformative changes to ensure sustainability. In particular, several participants highlighted the need for the NGO to have different sources of funding.

Further, many participants agreed that the government provided no support to the NGO during the COVID-19 pandemic:

*“We didn’t get anything from the government, and we had to buy all the supplies ourselves. We had to buy from the pharmacies and also from the pharmaceutical suppliers*” (P_9_ NGO Uganda Healthcare).

Other concerns related to the government’s approach to the COVID-19 pandemic emerged, including the lack of public health information available in different languages:

*The government tried their best to have charts with written information about COVID-19. But most information was not in our local languages, it was not translated, and it was in English. And most of our people here in Uganda, they don't know English, they use their local languages* (P_1_ NGO Uganda Field Officer).

One key avenue of support was international donors from the NGO’s partner organizations:

*The only support we received was the support from our partner organizations and those from the sponsors. Because at least the sponsors were concerned about what will happen to their seniors* (P_6_ NGO Uganda Village Volunteer).

## Discussion

This research presented the findings qualitative investigation of the strategies used by an NGO in Uganda during the COVID-19 pandemic to support older adults and how they are building back stronger from the pandemic. We found that the NGO used several innovative approaches to support older adults during the COVID-19 pandemic. Further, the findings highlighted the strategies NGO staff and volunteers have recommended to build back stronger from the pandemic.

NGO staff and volunteers utilized various strategies related to coping capacity, which addressed the immediate needs of the NGO and the older adults they support. For example, the NGO mobilized Village Health Teams to support older adults in the villages. Similarly, a non-profit based in Hong Kong used WhatsApp group chats to mobilize volunteers to provide PPE and other materials to vulnerable populations within their service networks.^35^ Technology has also been utilized to increase the efficiency of operations. Other NGOs, especially those in higher-income countries, have used electronic resources to inform the older adult population about public health measures.^36,37^ Another example of adjusting delivery methods is encouraging employees to work from home when possible. Work-from-home policies were utilized worldwide to slow down the spread of the virus.^38,37,39^ Overall, this highlights the importance of adjusting service delivery during unprecedented times and using available resources. It is essential to underscore how the NGO staff and volunteers were able to encourage a high percentage of older adults to receive the COVID-19 vaccine despite high levels of vaccine misinformation and hesitancy within the general population in Uganda and Ethiopia.^40,41^ In Uganda, for example, only 43% of the population received at least one dose.^42^ The vaccine discrepancy rate between the older adults at the NGO and the general Ugandan population underscores the importance of involving trusted community organizations in public health campaigns, which is a great example of adaptive capacity.

In contrast to coping capacity strategies, adaptive capacity focuses on using crucial learnings that can be used to protect against future public health crises. For example, the NGO has focused on strengthening healthcare facilities and continuing to focus on public health education. NGOs have always played a significant role in improving social health and public health in areas such as control of smoking, educating communities about infectious diseases, prevention of malaria and improving women’s health.^43^ Hence, the importance of NGOs cannot be understated. In terms of building back stronger, another area that was highlighted was the importance of having a reserve fund. A comprehensive report highlighted the importance of reserve money, given that those with reserve money were less likely to be negatively impacted by the COVID-19 pandemic.^44^ Another idea that can be brought to the forefront is the importance of collaboration between other NGOs and governmental sectors. NGOs can work closely with communities and can provide insights other sectors, such as government, cannot.^45^ While there are several challenges associated with public-private partnerships, such as a lack of mutual trust and interference from foreign donors,^10^ studies have highlighted the benefits of these partnerships. For example, in Ghana, the Ghana Health Service has collaborated with various NGOs to improve health outcomes in areas such as maternal mortality, sexual and reproductive health services and mental health.^46,47^ Research has shown that the collaborative partnerships between the state and NGOs led to improved service delivery and lowered health inequities.^47^ In Kenya, a public-private partnership led to an improved response to the COVID-19 pandemic by increasing COVID-19 testing capacity.^48^ By collaborating with a coordinating NGO, the initiative achieved scalability and improved efficiency, offering a model that can serve as a blueprint for future public health crises.^48^ Overall, these examples highlight the benefits of public-private partnerships when tackling public health crises.

Building on adaptive capacity measures, transformative capacity aims to create systemic change. A common theme underscored by the NGO was the donations and support received from international entities. While participants did not state whether funding from international donors increased or remained consistent, it is important to note that in the literature, several studies showed that funding and donations did decrease during the pandemic.^49,37,50^ For example, a comprehensive report stated that in Africa, over 50% of the 1015 community-based organizations surveyed stated that they had a loss of funding, with 69% of them having to cancel or reduce their services.^49^ One key theme within this study was that NGO staff and volunteers wished to receive more support from the government during this time, which was a common finding in other countries. For example, within the United States of America, due to the failure of the government to implement a strong response to the pandemic, many community organizations had to manage the public health response.^38^ These findings highlight the need for governments to assist NGOs in providing services to the community as NGOs can help bridge the gap between the government and grassroot levels.^12^ One way this can be done is for the government to set up care funds for NGOs as a buffering solution to help mitigate the impacts of another public health crisis.^51^ Similarly, diversifying revenue streams is another recommendation that can mitigate the vulnerability of the NGO and strengthen the financial health of the organization.^52,53^ While the majority of the revenue NGOs in the Global South receive is from international sources, other examples include grants from agencies, domestic government, membership, fundraising and donor revenue.^54^ Specific examples of successful diversification strategies include BRAC, which has focused on partnering with governments and other foundations to engage in long-term, multi-sector, multi-programme initiatives.^55^ Kenya’s Red Cross Society established commercial ventures such as a private ambulance and a chain of hotels to help generate domestic income.^56^ Finally, SOS Children’s Villages, including the location in Nigeria, engages in partnerships with corporate organizations to secure funding and in-kind support, which helps to enhance their financial sustainability.^57^

## Limitations

Study limitations were few but important; first, at the time of the interviews, the COVID-19 pandemic and Ebola cases were a concern, making it difficult to interview older adults. Further, both recall bias and social desirability bias may have impacted the respondents’ answers as individuals may want themselves and the NGO to be seen in a favourable light. Recall bias may have played a role as the interviews were conducted after the height of the COVID-19 pandemic. Hence, individuals may have had difficulties remembering specific events or unintentionally emphasized certain aspects of their experiences during the COVID-19 pandemic. Additionally, for future iterations of this research, it is crucial to include the perspectives of older adults to understand their experiences with the strategies implemented by the NGO and identify areas for improvement in response to future public health crises. It would be crucial to also see how the COVID-19 response compares to other public health crises that may have occurred during their lifetime. By doing so, best practice guidelines can be developed by incorporating the experiences of individuals who have lived through various crises. Another key step would be to incorporate other NGOs within this research to better understand the strategies they used during the COVID-19 pandemic and how they are recovering. For example, staff and volunteers from other NGOs in SSA, such as COHESU in Kenya, could be interviewed to understand how they navigated the pandemic and the strategies they recommend. This approach would not only help further develop the social resilience framework within this field of study, but also create a toolkit that can be shared with other NGOs and government agencies to better prepare for future public health crises.

## Conclusion

**Figure 1.**
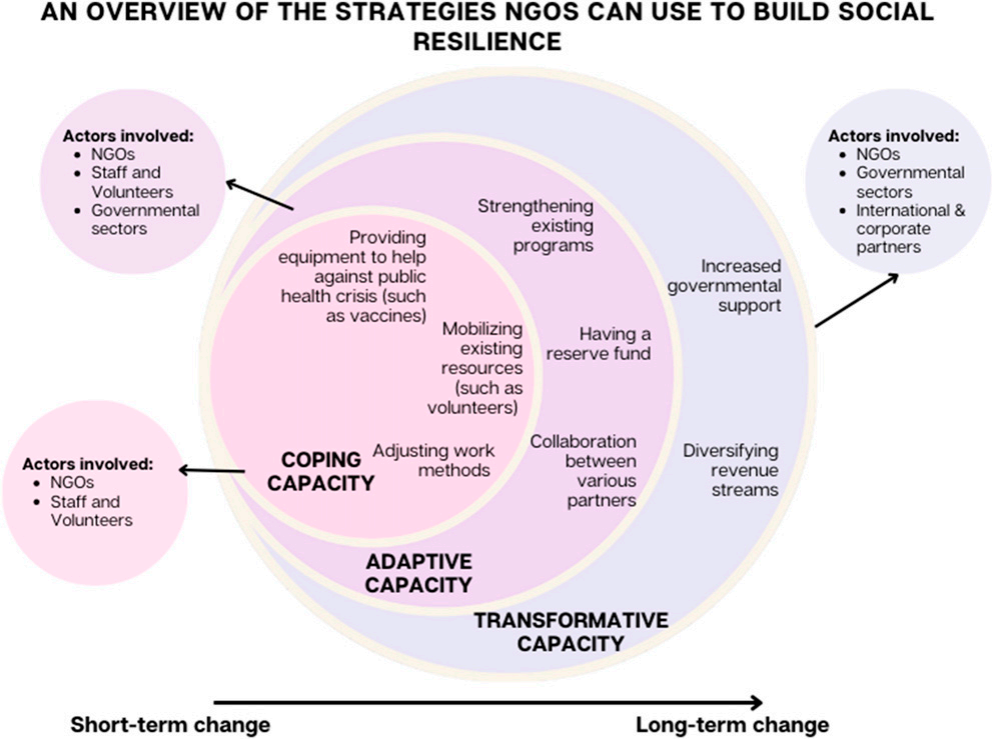
An overview of the strategies NGOs can use to build social resilience.

Our study focused on the strategies used by an NGO in SSA to support older adults during the COVID-19 pandemic and build back stronger. Through the social resilience framework, we identified several strategies related to providing support to vulnerable communities during public health crises and how NGOs can become more resilient for future crises (see Figure 1). Examples of these strategies included creating Village Health Teams to bring healthcare services closer to older adults, implementing a reserve fund, utilizing COVID-19 public health measures to protect against other public health crises and receiving international support.

Our research highlighted the experiences of NGOs during an unprecedented public health crisis. Key recommendations that can be used by other NGOs and other relevant actors is the importance of the government partnering with trusted community organizations to help disseminate information and resources, the need for governments to provide more resources to NGOs and how NGOs can become more resilient through adjusting service delivery and funding methods. To highlight, NGOs can focus on diversifying revenue streams and partnering with other actors to enhance sustainability and impact. These recommendations can be implemented in various contexts across SSA to strengthen the social resilience of NGOs, enabling them to continue to create innovative strategies to help vulnerable populations.
